# Dynamic cognitive inhibition in the context of frustration: Increasing racial representation of adolescent athletes using mobile community-engaged EEG methods

**DOI:** 10.3389/fneur.2022.918075

**Published:** 2022-12-21

**Authors:** Caitlin M. Hudac, Jessica S. Wallace, Victoria R. Ward, Nicole R. Friedman, Danae Delfin, Sharlene D. Newman

**Affiliations:** ^1^Department of Psychology, University of South Carolina, Columbia, SC, United States; ^2^Center for Youth Development and Intervention, University of Alabama, Tuscaloosa, AL, United States; ^3^Department of Psychology, University of Alabama, Tuscaloosa, AL, United States; ^4^Center for Autism and Neurodevelopment Research Center, University of South Carolina, Columbia, SC, United States; ^5^Department of Health Science, Athletic Training Program, University of Alabama, Tuscaloosa, AL, United States; ^6^Alabama Life Research Institute, University of Alabama, Tuscaloosa, AL, United States

**Keywords:** cognitive inhibition, frustration induction, electroencephalography (EEG), community-engaged research, brain injury, N2 component

## Abstract

**Introduction:**

Concussive events and other brain injuries are known to reduce cognitive inhibition, a key aspect of cognition that supports ones' behaviors and impacts regulation of mood or affect. Our primary objective is to investigate how induction of negative affect (such as frustration) impacts cognitive inhibition and the dynamic process by which youth athletes modulate responses. Secondary objective is to address the lack of Black representation in the scientific literature that promotes brain health and investigates pediatric sports-related brain injury. In particular, neuroscience studies predominantly include White participants despite broad racial representation in sport, in part due to technological hurdles and other obstacles that challenge research access for Black participants.

**Methods:**

Using electroencephalography (EEG), we evaluate the dynamic brain processes associated with cognitive inhibition in the context of frustration induction in adolescent athletes during pre-season conditioning (i.e., prior to contact; *N* = 23) and a subset during post-season (*n* = 17).

**Results:**

The N2 component was sensitive to frustration induction (decreased N2 amplitude, slower N2 latency), although effects were less robust at postseason. Trial-by-trial changes indicated a steady decrease of the N2 amplitude during the frustration block during the preseason visit, suggesting that affective interference had a dynamic effect on cognitive inhibition. Lastly, exploratory analyses provide preliminary evidence that frustration induction was less effective for athletes with a previous history of concussion or migraines (trending result) yet more effective for athletes endorsing a history with mental health disorders.

**Discussion:**

We emphasize the urgent need to improve representation in cognitive neuroscience, particularly as it pertains to brain health. Importantly, we provide detailed guides to our methodological framework and practical suggestions to improve representative participation in studies utilizing high-density mobile EEG.

## Introduction

Adolescence marks a critical period in which youth may be particularly susceptible to long-term effects of brain injury that includes concussion (1, 2). Brain development during adolescence involves an imbalance between the rapid growth of reward-seeking subcortical regions with the relatively slow development of the prefrontal cortex that broadly supports executive functions, such as cognitive inhibition, attention, and memory (3–5). If not properly treated, exposure to repetitive head/brain injury from contact sports has been associated with neurological disorders that can manifest in later-life, including chronic traumatic encephalopathy and other neurodegenerative diseases (6).

Within the United States, access to equitable on-field medical care for concussion is often a barrier in adolescent sporting contexts (7) and access is more often lacking in lower socioeconomic communities (8). Social factors that include pathways between where and how people live and access to medical care fundamentally affect health, including brain health, given that Black individuals are disproportionately more likely to live in lower socioeconomic communities (9). Thus, Black individuals are disproportionately more susceptible to health inequities relevant to head/brain injury that can create and reinforce brain injury health disparities (10, 11). Black American adolescent athletes are more likely to lack access to concussion education, are less likely to report symptoms or receive proper care and treatment and are more likely to suffer greater consequences through the life span (10, 11). Black athletes constitute 40–60% of eligible participants in American football, a high concussion risk sport (12). Despite the risk, Black athletes attending schools within urban communities, often of title I status, lack the appropriate access to health care professionals such as an athletic trainer who is trained in concussion education, recognition, and management (10). Racial and socioeconomic differences in concussion education, access to care, and appropriate management may negatively affect health outcomes for Black adolescent athletes.

Investigating how changes in cognitive abilities following injury may impact affect (emotion) regulation is an area of study that is lacking, both broadly in the literature as well as a focused target on health inequities for Black adolescents. There is increasing evidence of emotion dysregulation following brain injury (13, 14), in part due to disruptions of cognitive inhibition that supports the capacity to regulate and manage mood or affect (15). Prior work provides evidence of long-term problems with inhibitory control and cognition following concussion and head injury (16–19). This may be one reason why long-term affective and emotion regulation difficulties are also experienced (20). This is a critical area of concern given the relevance of affect regulation in support of positive well-being and quality of life (21, 22) and, consequently, improving affective/emotion regulation has been a subsequent area for intervention in concussion and brain injury (23, 24).

One of the difficulties with examining affective interference is methodological: how do you appropriately induce and track changes in cognition in the context of affect? First, it is important to utilize a well-known task that captures a robust cognitive marker and can examine state-level changes when affect is manipulated. One existing paradigm utilizes a common cognitive inhibition task wherein participants respond as quickly as possible to “go” stimuli and must inhibit a response to “No-Go” stimuli (25, 26). Behavioral performance indicates reduced accuracy to inhibition (“No-Go”) following a brain injury (27, 28), which reflects difficulty in suppressing or fully inhibiting prepotent responses (i.e., dominant response over other potential responses). Other researchers have adapted this task to frustrate participants implicitly by varying the speed and performance feedback (29, 30), subsequently providing a unique opportunity to track how cognitive inhibition (as measured by performance or brain function) is influenced by affect.

Second, it is important to consider instantaneous and ongoing dynamic changes during emotion regulation using cognitive neuroscience techniques (31). Electroencephalography (EEG) captures scalp potentials that map on to discrete states of cognition (32) and can be used to track state-level affective changes. For instance, after being frustrated in the affective Go-NoGo task (29), adolescents show a drastic reduction in the N2 amplitude. The N2 is a negative deflection occurring ~250 milliseconds (ms) across frontocentral electrodes thought to be elicited by the anterior cingulate cortex in response to inhibiting the prepotent response (33). Previous evidence indicates that adults with mild traumatic brain injury exhibited a reduced NoGo N2 responses when primed by a negative affective symbol (e.g., spider versus flower, 33). The N2 is likely a relevant and important target, as evidenced by reduced N2 amplitudes for young adults with a history of concussion during other cognitive tasks (34, 35). However, despite the need to examine the instantaneous dynamic changes (36), no work has utilized a trial-level analytic approach to understand how increasing frustration influences cognition on a moment-to-moment basis during this task.

### Current study objectives

We have two objectives in the current study.

Our primary objective is to examine *dynamic* changes in cognitive inhibition in the context of affective interference when frustration is induced. In this longitudinal study (two time points, before and after the season), participants completed the affective Go-NoGo task in which the rules implicitly shifted over the course of the experiment to induce frustration during the middle block when participants begin losing points at an accelerated rate (30, 37). In this way, we tracked how brain responses (e.g., N2) are modulated during baseline and frustration, as well as whether responses recover when the original rules are restored. Because we implemented a single-trial strategy, we measured the changes when frustration is induced, as well as the moment-by-moment dynamic changes across the task. In response to frustration, we predicted changes in N2 amplitudes to reflect changes in cognitive effort, examined as both an overall block effect (i.e., averaged N2 amplitudes as a main effect) and as a dynamic effect (i.e., slope differences within a block). Thus, we predicted that interference from heightened affective experience (i.e., when frustrated), cognitive inhibition would be reduced, as reflected by less negative N2 amplitudes. We focus on amplitude because latency has not been previously measured in these contexts; however, we posited that this competition would also relate to slower cognitive response (i.e., increased N2 latencies) and present these results in supplemental information. Lastly, we predicted that the frustration induction would increase *within* each block, such that frustration N2 amplitudes would habituate (i.e., become less negative). As an exploratory analysis, we also planned to test individual differences based upon history or symptoms of (a) concussion and (b) mental health disorders. Based upon studies that used the Go-NoGo paradigm in adults with a history of brain injury (38) and adults with major depression (34), we predicted that N2 amplitudes would be further reduced during frustration for adolescents with a history of concussion or mental health disorders.

Our secondary objective is to address the lack of Black representation in the literature, including head/brain-injury studies, as well as cognitive neuroscience broadly. Despite the prevalence of Black adolescent athletes [46–64% play at least one sport; (39)], Black individuals comprise < 29% of study samples (40) or race is not reported at all (41). Often research is centralized within university settings, especially studies of brain function; however, there are many obstacles that may prevent Black adolescents from engaging in settings. Barriers to research participation for Black adolescents may stem from the scientific overuse of passive recruitment strategies (e.g., digital or paper fliers), difficulties with access (e.g., transportation, poor health care presence), or other psychological and practical aspects of being historically excluded from research.

A particular problem is that brain imaging equipment and procedures are often ill-equipped for Black participants. For instance, EEG requires electrodes to record brain activity to be close to the scalp, yet the equipment was not developed to accommodate coarse and curly hair textures that push against the electrodes. Since these hair textures are common in Black participants, many previous studies have regrettably excluded these participants. Importantly, many initiatives have begun to address this systemic issue to improve Black inclusion in science (42) and reduce bias in neuroscience (43, 44), including development of novel electrode types for coarse and curly hair (45). Within the current study, we have specific reasons for using our existing high-density “wet” EEG system (most notably ease of application for sensory sensitive samples in our other work); however, we detail our procedures and developed resource guides for other users of this system. In addition, we describe the methodological framework implemented in the current study to serve as a guide with practical recommendations to improve diverse participation. We detail specifics pertaining to community-engaged research, including recommendations for starting and maintaining engagement and improving access.

## Materials and methods

### Participants

This study is part of a larger study on concussion and health-related quality of life led by J.S.W. Enrollment was offered to all adolescent high school American football athletes between 13 and 18 years old at one local high school (*N* = 52). The entire football team was approached for recruitment; however, to be eligible to enroll in the study, participants had to return a parent consent. Within the current study, participants completed an additional battery of EEG tasks along with measures for the larger study before (Visit 1) and after season (Visit 2) contact play. This high school continues to be involved in the ongoing research; however, this study solely focuses on EEG data collected during the 2021 season. Here, we analyzed data from the 23 athletes that completed the EEG tasks during preseason conditioning (i.e., prior to contact in practice or games). A subset (*n* = 17) of the athletes returned during postseason team activities to complete a follow-up EEG (Visit 2). Demographic characterization is provided in Table 1. In alignment and collaboration with the school district, the local ethical review board approved this project and procedures. All participants gave written informed assent and a parent or guardian provided written informed consent.

**Table 1:** Demographic information.

	**Visit 1**	**Visit 2**
*N*	23	17
Age Mean (SD) in years	16.13 (1.29)	15.86 (1.29)
Free-and-reduced lunch eligible *n* (%)	22 (95.7%)	13 (92.9%)
**Health history** ***n*** **(%)**		
Diagnosed concussion	4 (17.4%)	1 (7.1%)
Suspected concussion	2 (8.7%)	2 (14.3%)
Headaches	11 (78.6%)	15 (65.2%)
Migraines	15 (65.2%)	10 (71.4%)
Motion sickness	4 (17.4%)	4 (28.6%)
Brain injury / Surgery	2 (8.7%)	0 (0%)
Vision problems	3 (13%)	1 (7.1%)
Anxiety	5 (21.7%)	4 (28.6%)
Depression	5 (21.7%)	3 (21.4%)
ADD/ADHD	6 (26.1%)	2 (14.3%)
**Hairstyle** ***n*** **(%)**		
Afro-textured hair	5 (21.7%)	4 (28.6%)
Braids or locks	6 (26.1%)	3 (21.4%)
Short-hair (e.g., short crop, buzz cut)	6 (26.1%)	3 (21.4%)
Twists	6 (26.1%)	6 (42.9%)

### Individual difference measures

At the preseason Visit 1, participants completed a battery of self-reported items of health history, including any history of diagnosed concussions, number of concussions experienced, headaches or migraines (i.e., concussion symptoms), mental health disorders (e.g., anxiety, depression, attention-deficit hyperactivity disorder), and vision abnormalities. Questions were binary (yes or no) and referred to any history of these health issues prior to the date of data collection. Full results to self-report health history are presented in Table 1. We utilized this self-report data to examine the possible role of a previous concussion, symptoms associated with concussion, and mental health on EEG dynamics. In this way, participants could endorse multiple features (concussion, migraines, mental health).

### Community-engaged methods

The central idea behind community-engaged research is that research exists via relationships between researchers, the community, and community leaders (46, 47). The foundation of this methodology includes active community participation that facilitates trust, buy-in, and visibility of the research team (48). In this way, research does not use passive recruitment strategies such as emails or digital or physical fliers. A community-engaged approach also go beyond simply collecting data, followed by community-based absence. Rather, this approach is based on a continual community-based presence and partnership that involves a co-production of knowledge and translation with membership and leaders.

This study was rooted in community connections already established by the research team with high school administrators, athletic trainers, coaches, parents, and athletes involved with high school football programs across the state of Alabama. Consistent visibility included bi-weekly visits and attendance at football games as a community-engaged partner. When the study was first introduced to the prospective participants, we requested permission and used ~10 min of a team practice to talk about the current research opportunity and led a hands-on demonstration of how the EEG nets are put on the head. We passed out study information guides for the athletes to bring home to their parents to review. We then spent several days attending practice to collect written informed consent and remained engaged as part of the team during practices. Results of this study will be shared with the participants and the community via multiple avenues, including brief video summaries and infographics.

### Electroencephalography (EEG) methods – mobile approach

Portable equipment was utilized to collect EEG data during preseason conditioning and postseason visits. A mobile approach to EEG testing was critical for engagement in the study as it reduced the burden of participation on athletes and their families, generated an opportunity for diverse athletes who historically have not been engaged in research, and created a centralized testing site in order to test multiple athletes at the same time. Equipment for testing included hardware (EEG amplifier, electrical isolator, hubs to connect separate devices), computers, supplies, and EEG nets. Our EEG nets are prefabricated with stretchable elastomer that ensures each electrode is in a predetermined location relative to other electrodes. Wires are bundled on the EEG net and connected to an adaptor that interfaces with the EEG amplifier. All equipment was transported in hard-shell protective cases to a local high school and set-up in an athletic training room in the stadium fieldhouse. Researchers worked closely with school staff to identify and select an area for testing that was easily accessible to athletes, but not currently in use. Some minor adjustments to the environment were made (i.e., locating additional tables and chairs) and close proximity to a sink for preparing and cleaning nets was an important consideration. Researchers created a “staging” area in the room next door to the testing space, where nets were applied and adjusted in advance of the session, as to maximize timing and efficiency of back-to-back testing. Laptops were used instead of desktop computers to display stimuli and record EEG data. Nets of various sizes were packed carefully with other supplies (e.g., potassium chloride, measuring tape, towels). Researchers arrived early at the testing site to make any needed adjustments to the environment and to allow time for troubleshooting any possible technical problems. A meter was used to ensure the area selected did not have electromagnetic field interference. Photos of the EEG set-up were taken to ensure consistency between testing days.

EEG procedures for net placement: Here, we describe our procedures for using Magstim-EGI Hydrocel geodesic sensor nets with 128-channels (electrodes), which is a high-impedance “wet” net system. Because this style of EEG involves prefabricated nets, electrodes are not placed one at a time but rather connected as one cap/net and all electrodes are placed for every session. Special considerations for net placement were taken to accommodate hair types (e.g., coarse and curly hair, Afro-textured hair) and styles (e.g., locks, braids, twists). We developed resources for the research community (Supplementary material I) that detail procedures we used to ensure successful participation of all participating athletes. Additional details are also available as demonstrations for future participants (Supplementary material II).

**Figure 1:**
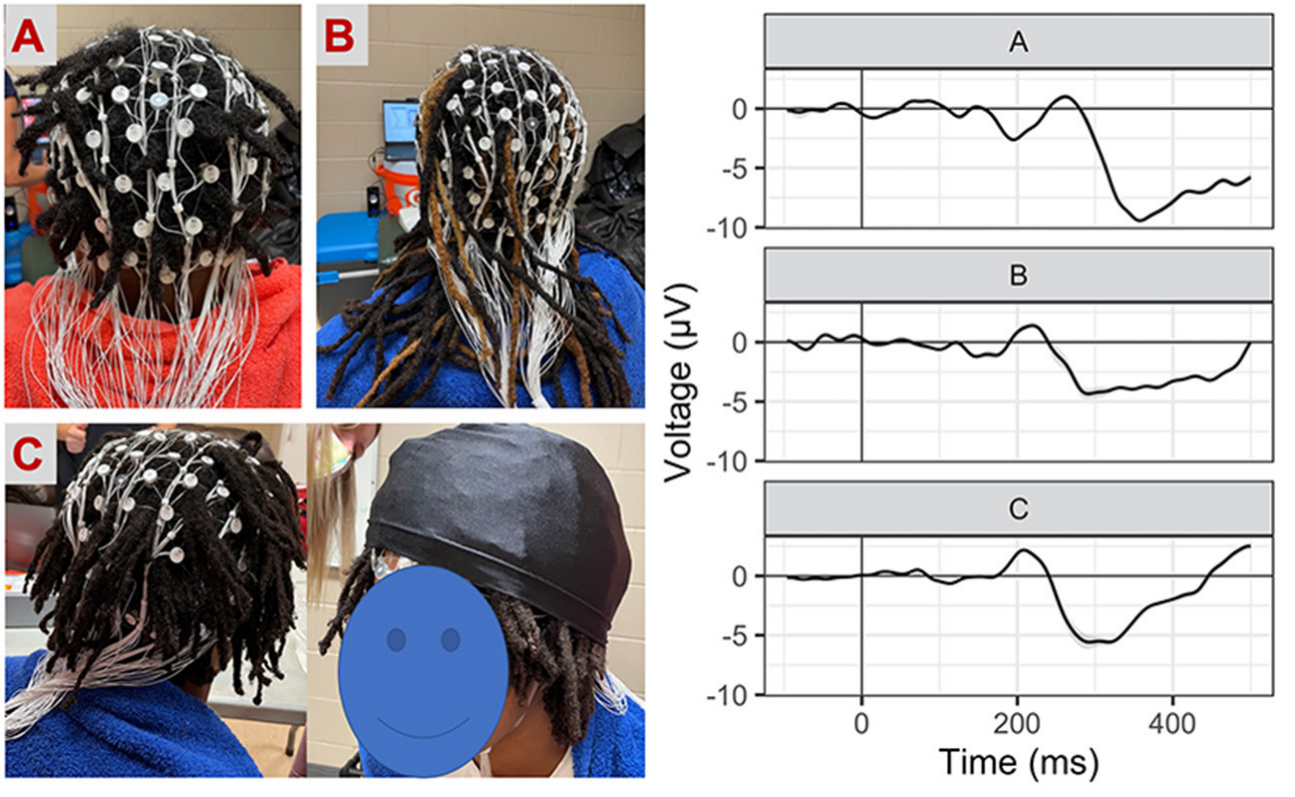
Net placement and signal quality examples. Grand-average waveforms to No-Go (i.e., inhibition) trials (collapsed across block) for three participants with different hairstyles [**(A)** twists, **(B)** long locks, **(C)** locks with wig cap].

1. Personnel: Each net was applied by two researchers. One researcher focused on primary net placement, while the second researcher stood behind the participant and assisted in ensuring the net successfully covered the back of the head. With participant assent, videos and photos of net placement were taken for each athlete for training purposes. When adjusting impedances, 2–3 researchers were often working simultaneously to wet and adjust electrodes efficiently, as an additional researcher monitored the computer and labeled electrodes still in need of additional water or adjustment.
2. Net sizing: Head circumference was measured as usual, but decisions in net size (i.e., sizing up or down) were influenced by hair style. For instance, for athletes with hair styles that were particularly wide or tall (i.e., thicker locks or long [>7 cm] Afro-textured hair), a decision was often made to size up so that the net would fit comfortably when placed on the head. In this study, net sizes ranged from 54 to 64 cm, thus requiring the largest net produced by the manufacturer (Magstim-EGI).
3. Hair style: For hairstyles with loose braids or twists, care was taken to pull individual braids through the elastomer weaving to get the net closer to the scalp. For braids tight to the scalp (i.e., cornrows), nets were adjusted (i.e., front to back or left to right) to get as many rows of electrodes down to the scalp in between braids as possible. For thicker hair types or styles, athletic tape and/or skull cap was placed on top of the net to improve the connection between electrodes and the scalp. Participant hairstyle data was collected (see Table 1) to track strategies for successful net placement, quality of subsequent data, and to aid in development of materials for future use in studies seeking to improve diverse participation.
4. Prioritized regions: At times where hair types or styles made it difficult to get all electrodes tightly down to the scalp, electrode regions designated to be most important for the task were prioritized for placement. It is common in EEG research to examine ERPs at a select scalp location (i.e., frontal regions for the inhibition task). If there is *a priori* scientific rationale to focus on a prioritized region, we recommend including descriptions about any modifications and explicitly describe signal quality within the manuscript (represented in the current study as Figure 1).
5. In-session flexibility: While we have specified these considerations here to encourage future research participation, we acknowledge that most accommodations were attempted and subsequently generated as a successful technique during the preseason testing. In other words, the preseason sessions provided an opportunity for learning in the moment, and we found that within-session flexibility was important. We logged and documented our techniques (e.g., net placement videos and images) and individual differences (e.g., hair style data, impedance records). This was helpful for us in evaluating the signal quality, preparing for postseason testing, and serving as de-identified demonstrations for future participants (Supplementary material II).

### EEG paradigm

This response inhibition task was identical procedurally to prior neuroimaging work (29, 30). Participants were presented a series of everyday objects (e.g., balls, cars, shoes) with a thick border and told to press a button when the border was green (“Go” condition), but not press (i.e., inhibit the prepotent response; “No-Go” condition) when the border was red (see Figure 1) (29). A cartoon character updated the score every 20–30 s and participants were told that scores were based on both accuracy and speed. Faces with a fearful expression were presented immediately following score updates. Face stimuli included male and female exemplars from the NimStim set (49) that were cropped in oval shape to remove distracting hair features. All faces were centered so that the eyes appeared in the same location. Participants were told that they did not have to press a button for faces. Error feedback was given as a visual “X” on the screen and a loud buzzer sound for trials with an incorrect, omitted, or late response. Athletes were told that this was a baseline test and if they improved their score during the post-season session, they would earn an extra prize in addition to the monetary compensation ($20 for participant for each EEG session; $20 for parent/guardian).

Three blocks were presented implicitly without stopping between blocks as Baseline, Frustration induction, and Recovery. Go and No-Go stimuli were presented adaptively such that stimulus presentation was modulated based upon performance (i.e., speeds up after correct response, slows down after incorrect response). Timing and scoring varied across blocks and were identical to the prior study (30). Baseline stimuli were presented at a moderate rate (800–1,150 ms) and increased in speed by 20 ms for correct responses (earning +3 points for Go trials and +7 points for No-Go trials) and slowed by 70 ms for incorrect responses (no points earned or lost). Points could only accumulate during the Baseline block; there was no punishment for incorrect responses. Frustration induction stimuli were presented more rapidly (600–950 ms) and increased in speed by 50 ms for correct responses and slowed by 50 ms for incorrect responses. Importantly, no points could be earned during the Frustration block. However, incorrect responses were penalized by −15 points. Lastly, the Recovery block timing was identical to the first Baseline block. All answers earned points during the Recovery block, including +1 point for incorrect responses, +15 points for correct Go trials, and +25 points for correct No-Go trials. A minimum of 25 No-Go trials were presented each block.

### EEG equipment and data processing

Stimuli were displayed on a 15.5” widescreen Lenovo IdeaPad L340 laptop using E-Prime 3.0 (Psychology Software Tools, Pittsburgh, PA). Participants were seated approximately 110 cm from the monitor such that Go-No-Go stimuli were subtended 4.9 × 8.5° of visual angle and face stimuli were subtended 4.9 × 3.9°of visual angle. Continuous EEG was recorded from a high-density 128-channel geodesic net using Net Station 5.3 software integrated with a 400-series high-impedance amplifier (Magstim-EGI, Eugene OR USA). As per common standards for wet-style EEG systems with a high-impedance amplifier, additional electrotype solution was added to the sponges, and electrodes were seated onto the scalp to improve impedances. In order to reduce participant burden, limits were set so that impedance adjustment took < 5 min or when all electrodes were below 200 kohms. Although we prefer starting impedances to be below 50 kohms to maximize signal-to-noise ratio, others using long-term recording have determined that < 200 kohms is sufficient (50, 51). During acquisition, EEG signals were referenced to the vertex electrode, analog filtered (0.1 Hz high-pass, 100 Hz elliptical low-pass), amplified, and digitized with a sampling rate of 250 Hz. Data was preprocessed within NetStation 5.4.

**Figure 2:**
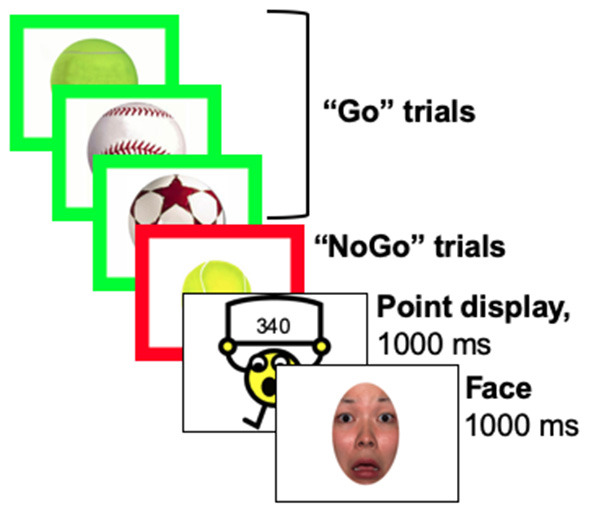
Stimulus presentation. Stimuli were presented adaptively based upon participant performance, such that the game increased in speed after correct responses and slowed down after incorrect responses. Speed changes and the points given or taken away changed across blocks, such that it became impossible to earn points during the frustration block and then impossible to lose points during the recovery block.

In this study, we focus our analyses on inhibition (e.g., “No-Go” trials). Standard post-processing procedures included bandpass filtering between 1 and 30 Hz, segmentation from −200 to 700 ms from stimulus onset, automatic blink and artifact detection and manual verification before bad channel replacement, baseline correction, and averaging within condition per participant. There were no concerns about signal quality regardless of hairstyle use and we illustrate some examples of individual subject waveforms in Figure 2. Here, we opted to focus on the frontal lateral N2 by averaging across electrodes^1^, as is customary in work examining cognitive inhibition via this task (29). Based upon visual inspection of the grand average waveform and aligned with prior work, the peak amplitude and peak latency for the N2 component were extracted from 225 to 500 ms post- No-Go stimulus onset. Latency results are available in supplemental material (Supplemental material III).

All analyses were performed using R (version 4.0.3). Linear mixed-effects models were computed using restricted maximum likelihood with Nelder-Mead optimization via the “lme4” package (52). Preliminary models indicated that neither amplitude nor latency were impacted by hairstyle, *p*'s> 0.22. Main amplitude results are reported within the main text but latency results can be found in Supplemental material. First, each model was fit with fixed effects of visit (preseason, postseason), block (baseline, frustration, recovery), and the interaction between visit and block. A random intercept was included for each participant to account for shared variance assumed with repeated measures. Bonferroni correction was used to adjust for multiple comparisons. Second, to assess cognitive dynamics within each block, additional models included a fixed effect of trial and all possible interactions with block and visit. Slopes were extracted for each block by visit using Johnson-Neyman techniques via the “jtools” package (53). For ease of qualitative description of each block, we opted to use a linear slope rather than test for potential slowing of the rate of habituation (e.g., as represented by a quadratic slope if the amplitude reached a plateau or steady state). Lastly, as an exploratory examination, we tested whether preseason (Visit 1) N2 amplitude and/or amplitude dynamics were modulated by subgroups that endorsed history of concussion, concussion symptoms, or mental health disorders. We elected to only use Visit 1 because it was the larger sample (*N* = 23) and was the only measurement point for the individual difference screener. Following the main analysis plan, in a series of models for each subgroup, a binary variable was added to the models such that the full factorial was assessed with fixed effects of visit, block, and subgroup (yes, no). Sample size was too small to test for intercurrent inclusion in different subgroups (e.g., concussion and mental health history) and is further described below in the limitations section of the discussion.

## Results

**Figure 3:**
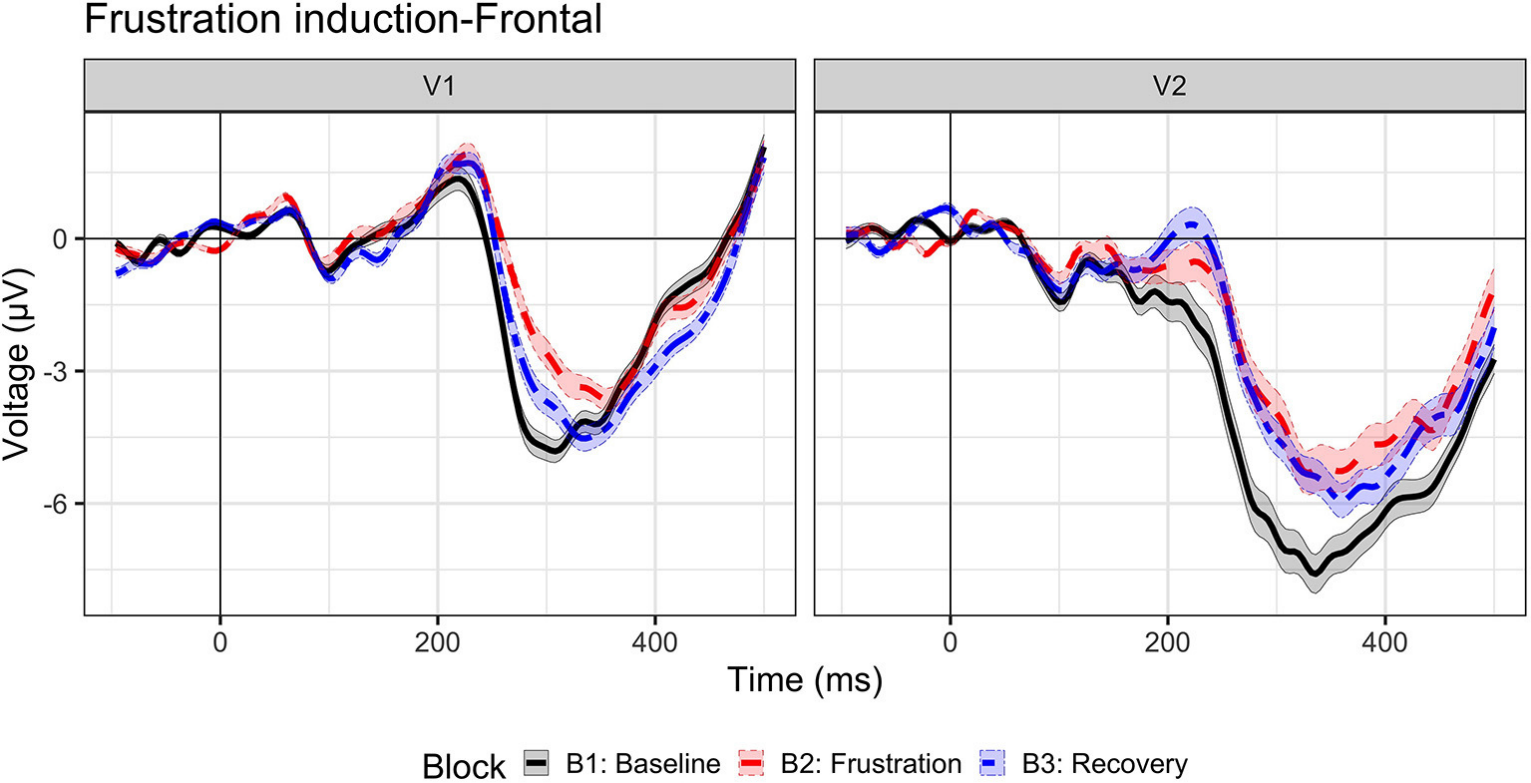
Event-related potential to inhibition (No-Go trials). Grand-average waveforms to No-Go (i.e., inhibition) trials for visit 1 (*N* = 23) and visit 2 (*n* = 17) by block: baseline (B1, black solid line), frustration (B2, red long dash line), and recovery (B3, blue dash-dot line).

Inhibition ERP waveforms (No-Go trials) are illustrated in Figure 3 for Visit 1 and Visit 2 across baseline, frustration, and recovery blocks. A frontal P2 was also present from ~150–300 ms but was not investigated in the current study.

### N2 amplitude

**Figure 4:**
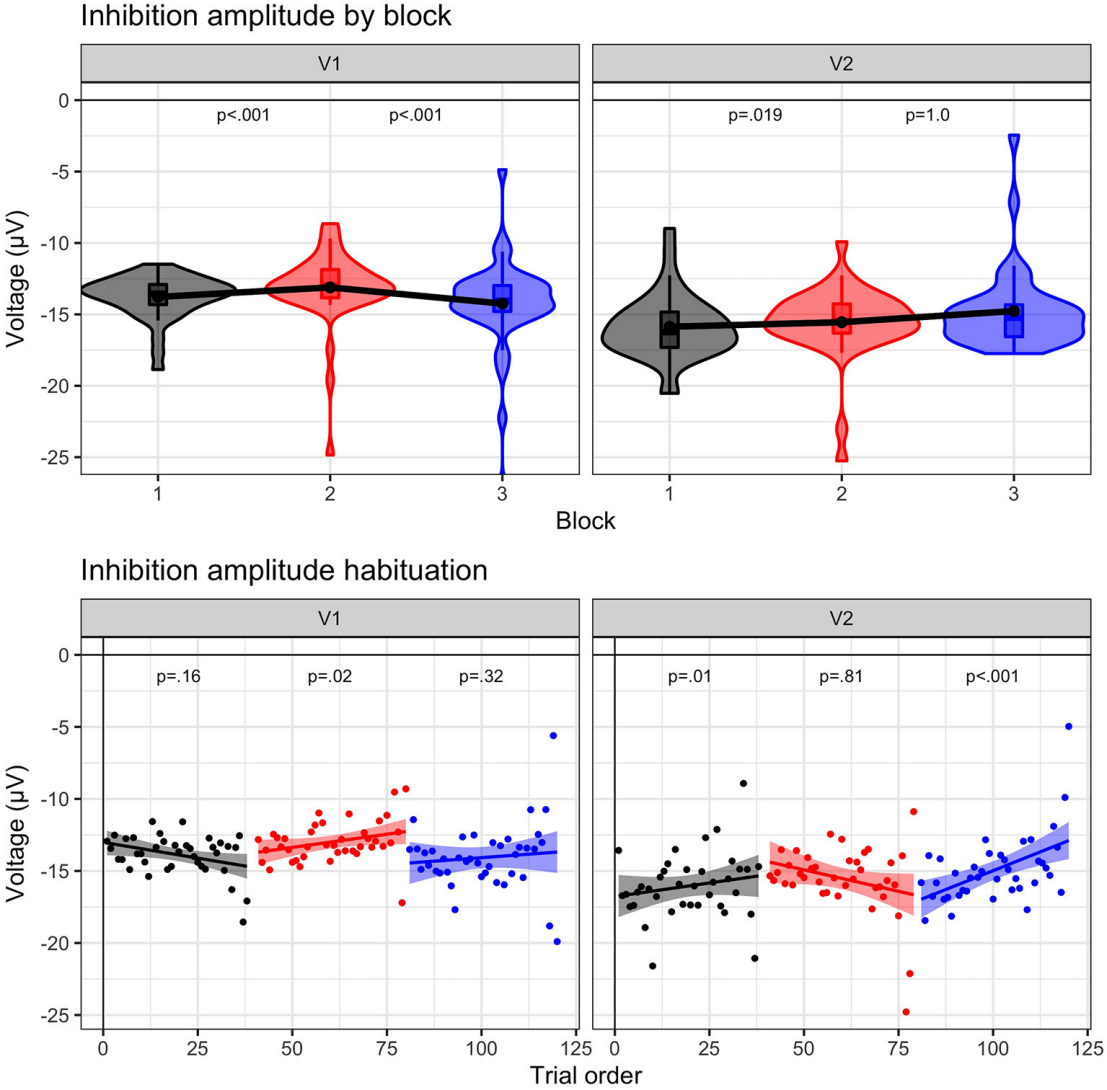
N2 amplitude results. N2 amplitude to No-Go (i.e., inhibition) trials for visit 1 (*N* = 23) and visit 2 (*n* = 17) by block: baseline (B1, black), frustration (B2, red), and recovery (B3, blue). **(Upper panel)** Violin plots indicate density of single-trial results, boxplots represent the quartile distribution, and the black dot and line connecting the three blocks highlight the mean value. Significance between blocks (B1 to B2; B2 to B3) is indicated. **(Lower panel)** Group-level trial averages are plotted across trial order for each block. Linear slope with 95% confidence intervals are plotted with significance value listed for each block.

N2 amplitude results are illustrated in Figure 4. These results suggest a distinct change in cognitive inhibition during frustration at both visits, aligned with our prediction that the N2 amplitudes would be reduced with heightened affect. A main effect of block, *F* (1.10546) = 19.06, *p* < 0.0001, indicated that N2 amplitude decreased (i.e., became less negative by ~0.82 μV) from baseline to frustration (B1 vs. B2, *p* < 0.0001) and returned to baseline values during recovery (B2 vs. B3, *p* < 0.0001; B1 vs. B3, *p* = 1.0). A main effect of visit, *F* (1.10546) = 126.5, *p* < 0.0001, indicated a more negative N2 amplitude at Visit 2, across blocks. The interaction between block and visit, *F* (2.10546) = 2.97, *p* = 0.051, indicated a trend, such that the block effect was prominent for Visit 1 (*p*'s < 0.0012); however, Visit 2 N2 amplitudes did not return to baseline levels (B2 vs. B3, p = 0.08, uncorrected).

### N2 amplitude dynamics

The results indicate that preseason and postseason visits exhibited different dynamic patterns during frustration. The three-way interaction between trial, visit, and block was significant, *F* (2.10542) = 4.28, *p* = 0.014. At Visit 1, only the frustration slope was significant (B2 slope = 0.03, *p* = 0.02), indicating that during the frustration context the N2 amplitude habituated (i.e., became increasingly less negative). In other words, trial-by-trial cognitive inhibition was maintained at equivalent levels during baseline and recovery but decreased due to affective interference during frustration. An opposite effect was observed at Visit 2. Both baseline and recovery slopes indicated habituation (B1 slope = 0.05, *p* = 0.01; B3 slope = 0.06, *p* < 0.001), but frustration slope was not significant (B3 slope, *p* = 0.81).

**Figure 5:**
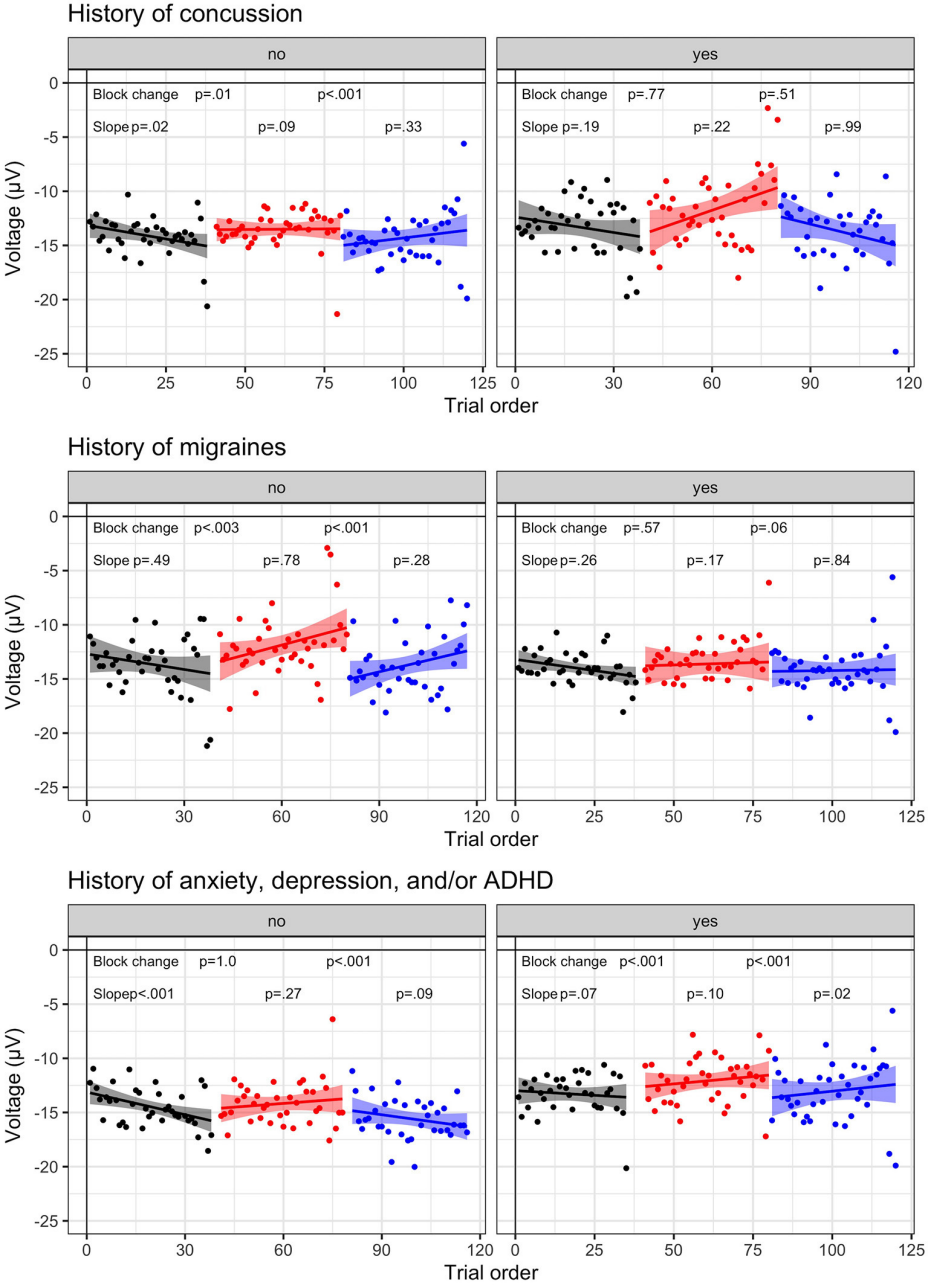
Individual difference N2 dynamic results. N2 amplitude to No-Go (i.e., inhibition) trials for visit 1 (*N* = 23) and visit 2 (*n* = 17) by block: baseline (B1, black), frustration (B2, red), and recovery (B3, blue). Group-level trial averages are plotted across trial order for each block for the following individual differences: History of concussion **(top row)**, History of migraines **(middle row)**, History of anxiety, depression, and/or ADHD **(bottom row)**. Left columns represent no history and right columns indicate endorsed history. Linear slope and 95% confidence interval is plotted with significance value listed for each block.

Influence of individual differences. Results with block change and slope significance are illustrated in Figure 5.

First, results suggest that cognitive inhibition is less impacted by affective interference in athletes with a history or symptoms of concussion. A trend indicated that block effects varied based upon a history of concussion, *F* (2.6510.8) = 2.45, *p* = 0.087, such that athletes who reported a previous concussion (*n* = 4 diagnosed; *n* = 2 undiagnosed) indicated a smaller change in N2 amplitude across blocks (*p*'s > 0.51, Bonferroni; *p*'s < 0.05, *uncorrected*). A similar trend was present for athletes reporting a history of migraines (*n* = 15), *F* (2.6510.9) = 2.99, *p* = 0.051. In contrast, athletes without concussion history or migraines exhibited strong block effects (*p's* < 0.01).

Second, athletes reporting a history of mental health disorders^2^ were more sensitive to the frustration induction (*p*'s < 0.001) than athletes without mental health history (*p*'s < 0.01), as indicated by an interaction between group and block, *F* (2.6510.7) = 5.98, *p* = 0.003. A three-way interaction between group, block, and trial, *F* (2.6511.8) = 3.96, *p* = 0.019, highlighted that athletes with a mental health history exhibited habituation (positive slopes >0.03), indicative of decreasing cognitive inhibition within each block. In contrast, athletes without mental health history exhibited negative slopes (B1 & B3, slopes < -0.04), suggesting that cognitive inhibition was increasing or remaining stable.

## Discussion

Our work is the first to examine the dynamic features of cognitive inhibition at two levels of time – block differences following a frustration induction, as well as trial-by-trial changes within each block. This study demonstrated that the frustration induction was successful: as a marker of cognitive inhibition, the N2 amplitude (i.e., strength of inhibition) became less negative and N2 latency (i.e., processing speed, see Supplemental material) became slower as athletes experienced affective interference. Prior neuroimaging work using the current task (30) demonstrated decreased amygdala activity (i.e., the primary emotion processing “gate” of the brain) with the frustration induction. The authors also found that amygdalar activation was modulated by the ventromedial prefrontal cortex activity, a key brain region supporting cognitive regulation of emotion (54, 55), as well as cognitive inhibition (56–58). There are developmental and maturational considerations for these findings, considering that the prefrontal cortex undergoes drastic structural and functional changes during early and throughout adolescence (59) that often oppose changes to network of brain regions supporting reward and affect/mood (60, 61). Although not explored within the current study, a deeper understanding of how pubertal maturation and/or age impact affective interference on cognition would be helpful.

The second level of cognitive inhibition dynamics utilized a trial-by-trial approach to understand ongoing changes within each block. Our results indicate that during preseason, as athletes became more frustrated, the N2 amplitude became less negative (i.e., habituated). This finding supports the hypothesis that affective interference would reduce cognitive inhibition, as previously indicated within clinical samples [e.g., dysphoria, schizophrenia; (62, 63)]. In contrast, the postseason N2 amplitudes exhibited the opposite pattern, such that baseline and recovery N2 amplitudes habituated. We are limited by only having two time points; however, there are several possible interpretations. These results could indicate test-retest issues on this particular task, wherein the manipulation was less impactful during postseason. There may be a novelty effect inherent in completing research procedures for the first time. Although not captured empirically, anecdotally, athletes were eager to improve their score at postseason to earn the extra gift card, and research assistants reported similar levels of affect dysregulation. To this extent, it may be helpful to evaluate the video recordings of the participants for facial displays of frustration. Alternatively, there may be other situational contexts that reduced the impact of affective interference at postseason. For instance, preseason testing was conducted prior to the start of the academic year during conditioning practices for football. The athletes were not currently engaged in academic work, and the research session was the first time many had completed a research study. By the postseason visit, the athletes were more familiar with the research environment and procedures.

Lastly, our individual difference results indicate preliminary support via a trend that the frustration induction was less effective (i.e., smaller block effects) in athletes with a history of concussion or concussion symptoms. This trending finding is aligned with other studies demonstrating a reduced N2 amplitude in groups with a history of brain injury (34, 35, 38). In contrast, the frustration induction was more effective in athletes that endorsed a history of anxiety, depression, and/or ADHD. Therefore, these individuals displayed greater difficulty in regulating their emotions during periods of frustration induction. This finding is expected as poor emotion regulation is considered a defining characteristic of diverse forms of psychopathology (64). Poor emotional regulation (i.e., emotion dysregulation) emerges as emotional processes become maladaptive and subsequently impede functioning. Emotion dysregulation, particularly of negative affect such as frustration, is posited as a cause of mood and anxiety disorders (65). Prior research suggests differences in N2 amplitudes during emotional induction among youth with ADHD and their typically developing peers (66), and emotion dysregulation has been implicated as a primary symptom in adult ADHD (67). Trial-by-trial data was not significant within these subgroups, likely due to the sample size, which we hope to increase in future projects.

### Important implications of community-engaged research methods

Our secondary objective was to address the lack of Black representation in the scientific literature that promotes brain health and investigates pediatric sports-related brain injury. We emphasize that mobile EEG testing, especially when combined with community-engaged methods, provides an opportunity to improve the representation of underrepresented populations. Predominantly Black high schools, as identified in our population here, are more often Title I schools that face resource limitations, commonly lacking access to healthcare professionals (8), education about brain injuries (10, 68, 69) and diagnostic tools used for concussion and treatment (68, 70). As a result of historical systemic racism, poverty disproportionately affects Black people compared to White people in the U.S. (71). Downstream effects of this contribute to marginalized communities being underrepresented in those enrolled in research studies and barriers to participation that can include working parents, transportation limitations, and trust in science or the healthcare system as a whole. Racial and socioeconomic differences exist in concussion awareness and attitudes (72), diagnosis (73), treatment, and outcomes (10, 74). Further, there are racial and socioeconomic disparities in performance on widely used computerized neurocognitive tests that are attributed to social determinants of health and a lack of cultural equivalence within the test (70). Systemic barriers such as the lack of concussion education and minimal-to-no access to trained health professionals within many urban and/or Title I schools puts adolescent athletes at a higher risk of having a concussion go undiagnosed and experiencing serious neurological symptoms throughout the lifespan. Mobile EEG testing does not solve the notable disparities, however, used in concert with established community-engaged methods, can advance its accessibility and utility.

### Future considerations and limitations

As the first study in this particular high school setting, there were several key elements that would be helpful to describe to improve success for future work (ours and others). First, due to the availability of the mobile system, our timespan for data collection was truncated to 4 days during pre-season and 7 days during post-season. We would prefer to have a longer window for participants to learn about the study, observe a demonstration, and retrieve a parental consent. Second, as acknowledged within the methods section, at the outset of this study (i.e., before Visit 1), we had limited resources prepared to describe our EEG procedures. For instance, during the preseason testing, we fine-tuned different net application procedures (e.g., pulling longer twists and locks through the EEG net webbing). In preparation of this paper, we have developed these resources for other researchers and for participants, which will be helpful for future efforts. Relatedly, although we had success with our *a priori* targeted cluster of electrodes to extract the N2 component, there were other electrode clusters that were further from the scalp due to thick hair. Other EEG electrode systems that are being developed may provide better full coverage by innovating the kind of electrodes used for coarse and curly hair (45). Third, the success for community-engaged research relies on continual engagement and presence in communities. It will be important for university and research sponsors to understand and support the costs and resources associated with these partnerships. For instance, current NIH policies restrict use of funds for food, which can have a direct impact in researchers' abilities to provide resources (for instance, snacks after practice) that support a mutually beneficial community-engaged partnership. Lastly, we acknowledge that EEG may not be useful for widespread clinical use, considering costs, accessibility, and training. However, there are clear scientific advantages, including elements used in this study, such as capturing momentary temporal dynamics. Current measures of cognition used for concussion rely on pencil/paper or computerized neurocognitive tools that provide insight into behavioral performance (e.g., reaction time, memory, processing speed), but not neural correlates or dynamic shifts in cognition. There are psychometric limitations of neurocognitive tools commonly used for concussion, as well as poorly embedded measures to detect low effort or sandbagging. Thus, this work contributes to a growing body of literature using EEG correlates to better track subtle cognitive changes following a concussion (75, 76).

## Conclusions

We addressed a gap in research related to the intersection of cognitive inhibition and affect regulation, with preliminary evidence showcasing differences following brain injury events. As a temporally precise technology, EEG provided a foundation to investigate the neural processes from a dynamic perspective, an advancement from prior behavioral tools. Cognitive inhibition was examined at two levels of time: block differences after frustration induction and trial-by-trial changes within each block. Frustration induction was successful as evidenced by changes in the N2 amplitude (marker of inhibition) and on a trial-by trial level N2 amplitudes habituated over time as expected during frustration. However, post-season testing revealed an opposite pattern, which may suggest test-retest issues with the task or otherwise highlight temporal relevance (i.e., before and during the academic year). Importantly, individual difference factors revealed less effective frustration induction in individuals with concussion history, and that athletes who endorsed a history of mental health disorders had a heightened response to frustration.

Despite widespread participation of Black athletes in sport, there is limited research addressing sports-related brain injury in this population. There are many barriers to research participation for Black adolescents such as overuse of passive recruitment strategies, difficulties with access, or other psychological and practical aspects of being historically excluded from research. In this study, it was critical to use community-engaged methods (48) that involved existing, established, and sustained community connections and active community participation with visibility that facilitates trust with the research team. A mobile EEG approach also reduced the burden of participation on athletes and their families and created a centralized opportunity for athletes often excluded from research. To address the need for more inclusive EEG research, methodological adaptations for coarse and curly hair were documented in an effort to create and provide resources for the research community as well as guidance and information for future participants.

## Data availability statement

The raw data supporting the conclusions of this article will be made available by the authors, without undue reservation.

## Ethics statement

The studies involving human participants were reviewed and approved by University of Alabama Institutional Review Board. Written informed consent to participate in this study was provided by the participants' legal guardian/next of kin.

## Author contributions

CH and JW contributed to the conception and the design of the study. CH, VW, and NF coordinated, managed EEG data collection, and compiled guides for researchers and participants. DD and JW coordinated and managed behavioral data collection. CH conducted data analyses and wrote the first draft of the manuscript. All authors contributed to manuscript revision, read, and approved the submitted version.

## Footnotes

^1^Sensor numbers on Magstim-EGI Hydrocel GSN sensor array included: Left frontal = 24 (F3), 27 (F5), 23, 33 (F7), 28, 34; Right frontal = 3, 116, 117, 122 (F8), 123 (F6), 124 (F4).

^2^See Table 1. Here we collapse across n = 12 who reported the following mental health issues: *n* = 5 with ADHD, *n* = 2 with ADHD and depression, *n* = 1 with depression, *n* = 2 with anxiety, *n* = 3 with anxiety and depression.
