# Supplementary material for: Dynamic cognitive inhibition in the context of frustration: Increasing racial representation of adolescent athletes using mobile community-engaged EEG methods
Source: Front Neurol. 2022 Dec 21;13:918075. doi: 10.3389/fneur.2022.918075 (PMC9812645; doi:10.3389/fneur.2022.918075)
Supplement: Supplementary file 1 [file Presentation_1.pdf]

EEG PARTICIPANT GUIDE

FOR INDIVIDUALS WITH COARSE AND CURLY HAIR

B-RAD Lab

What is EEG?

Electroencephalography (EEG) measures electricity from your brain as you think and move -- brain waves!

Why is it wet?

We use an EEG system that uses a net-like cap. We soak it in water to help record brain waves.

Examples of how EEG works on coarse & curly hair:

AFRO-TEXTURED

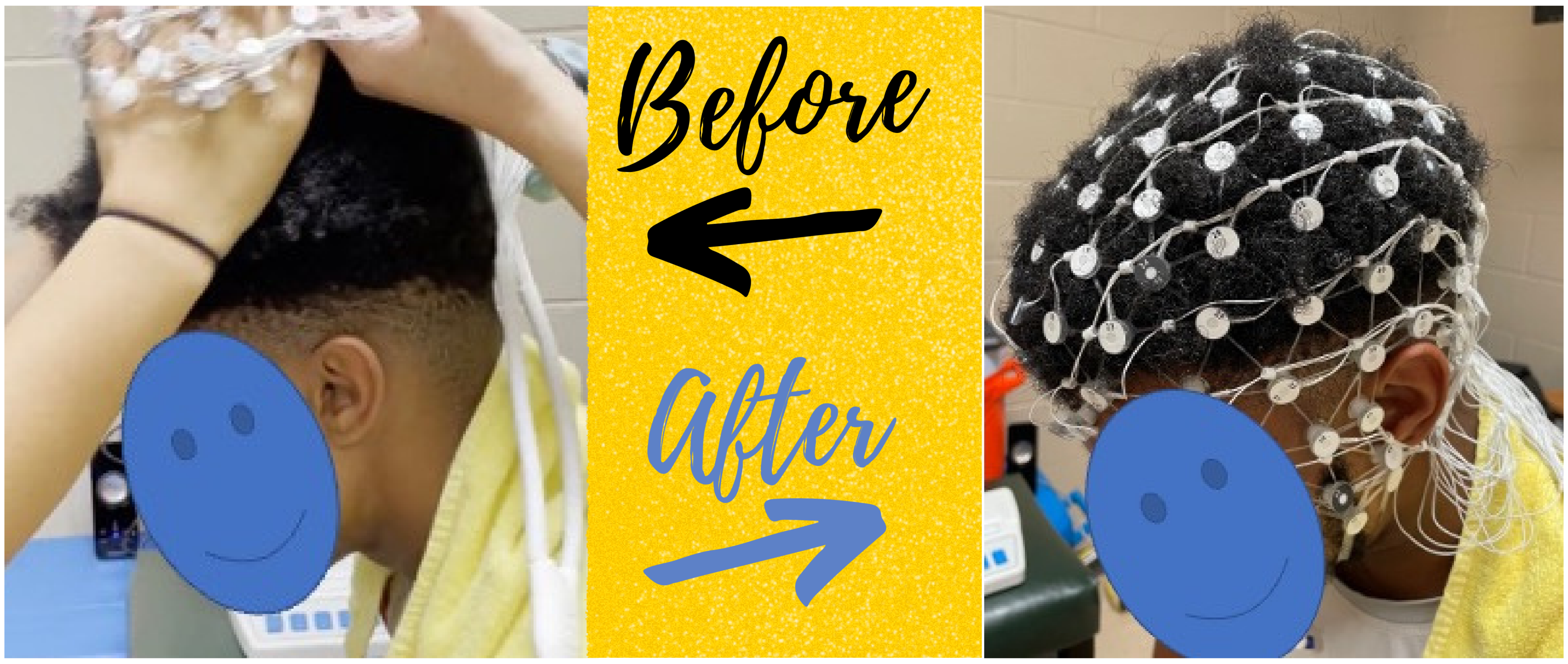

LOCKS

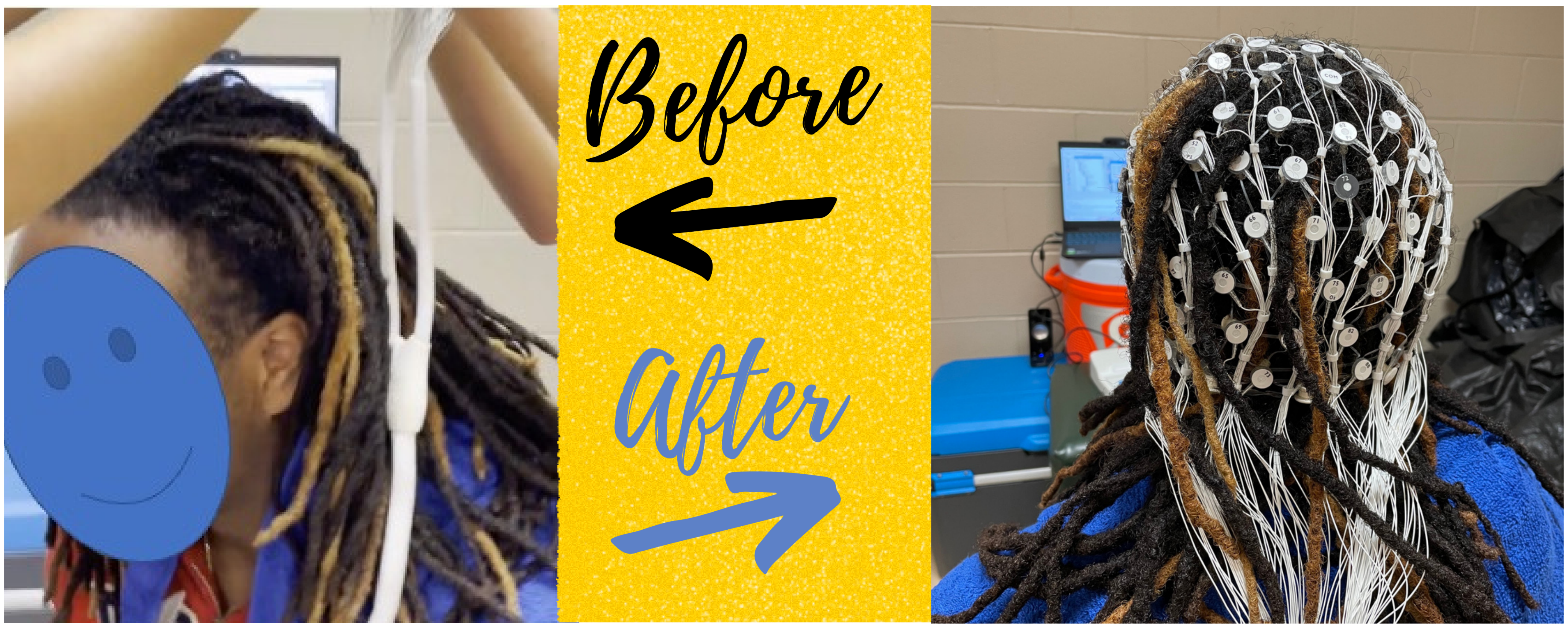

EEG PARTICIPANT GUIDE

More examples of how EEG works on coarse & curly hair:

BRAIDS & TWISTS

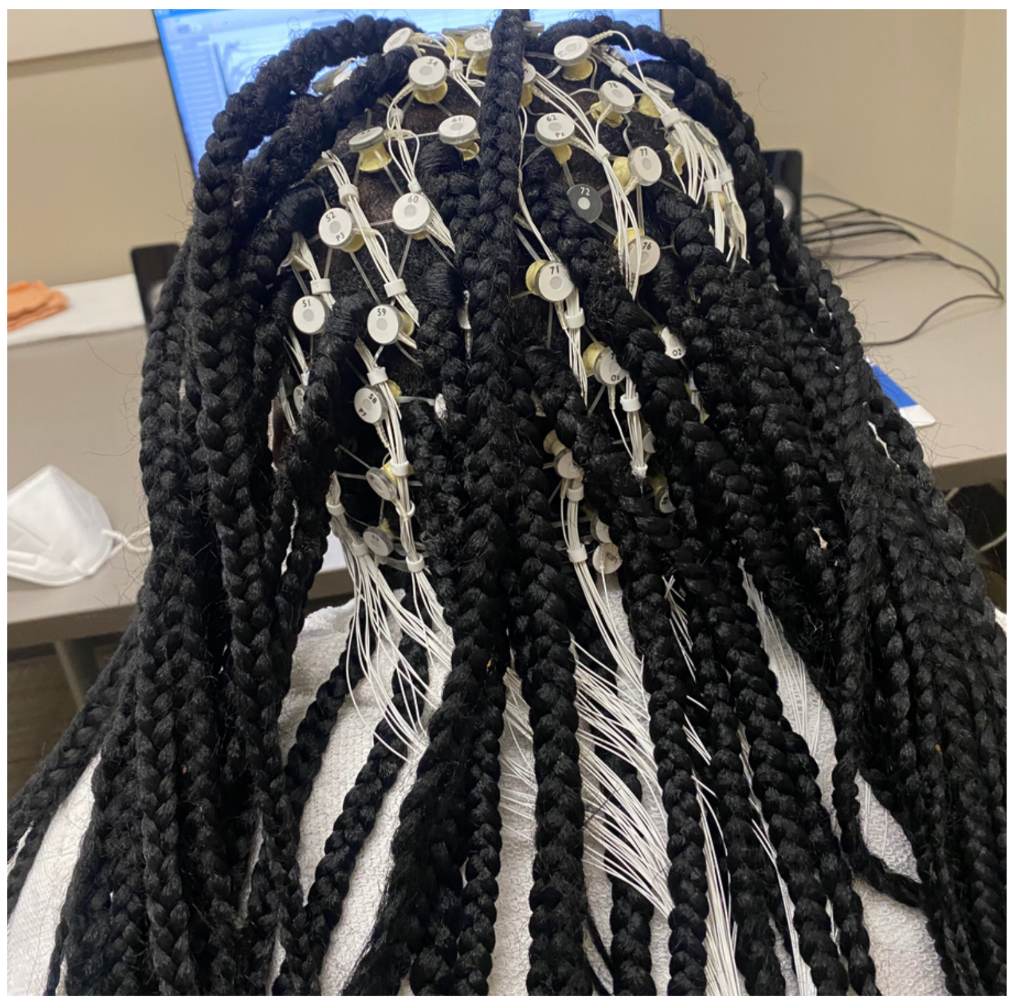

We pull the braids through the holes in the EEG net

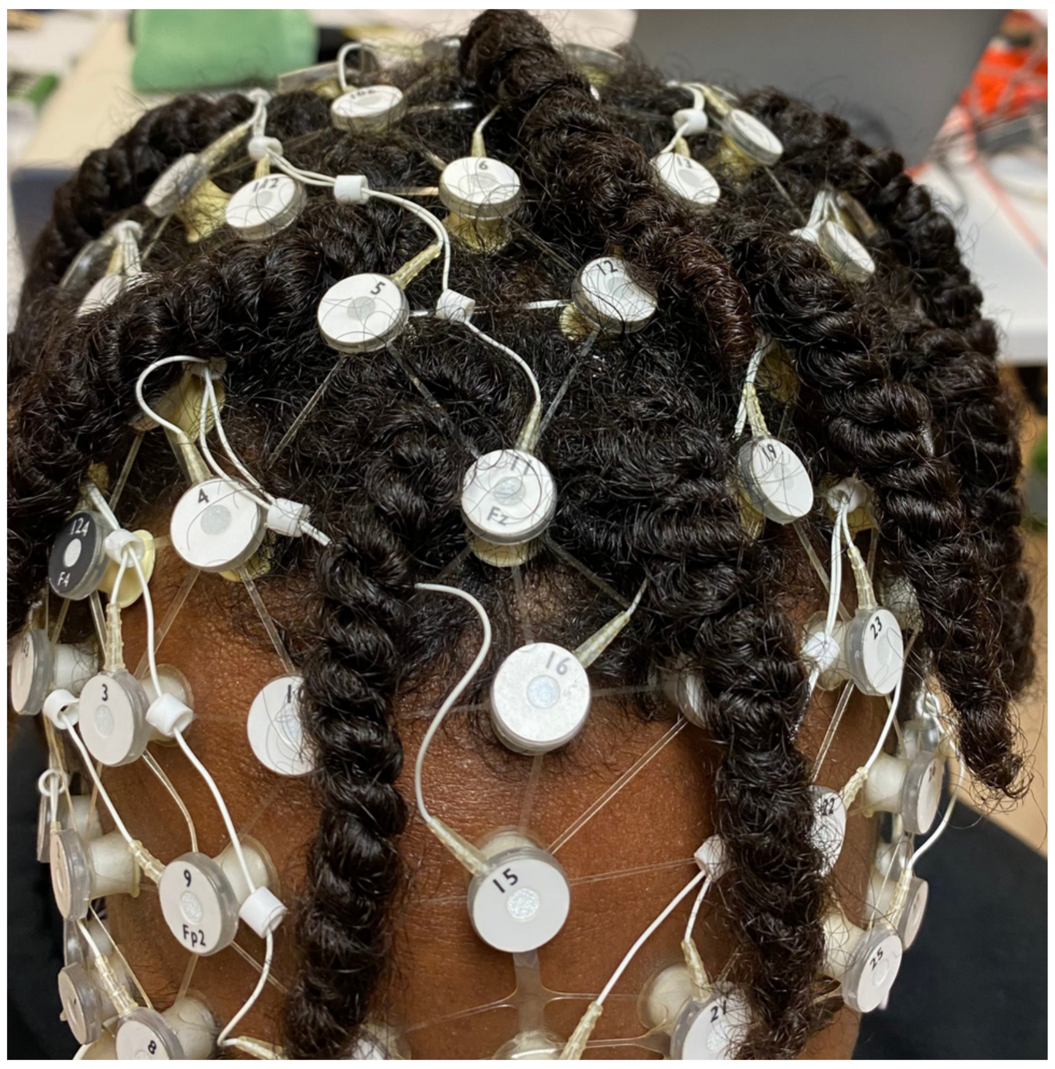

Lace WIGS *& more*

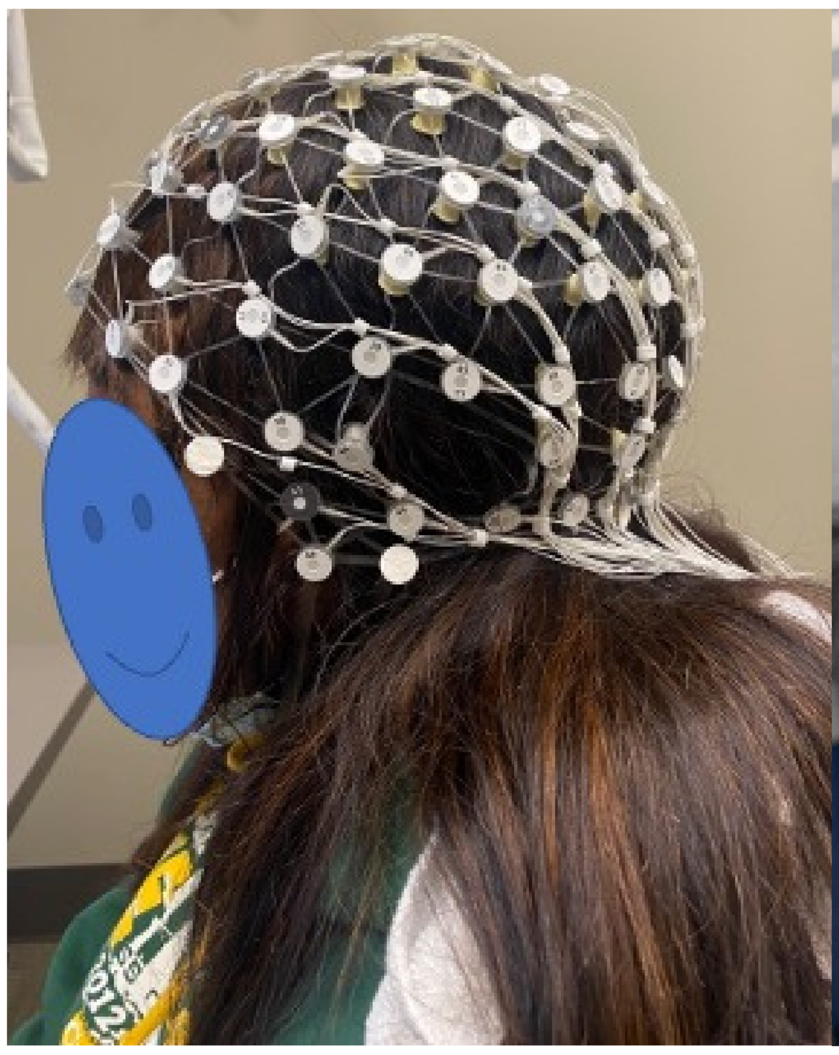

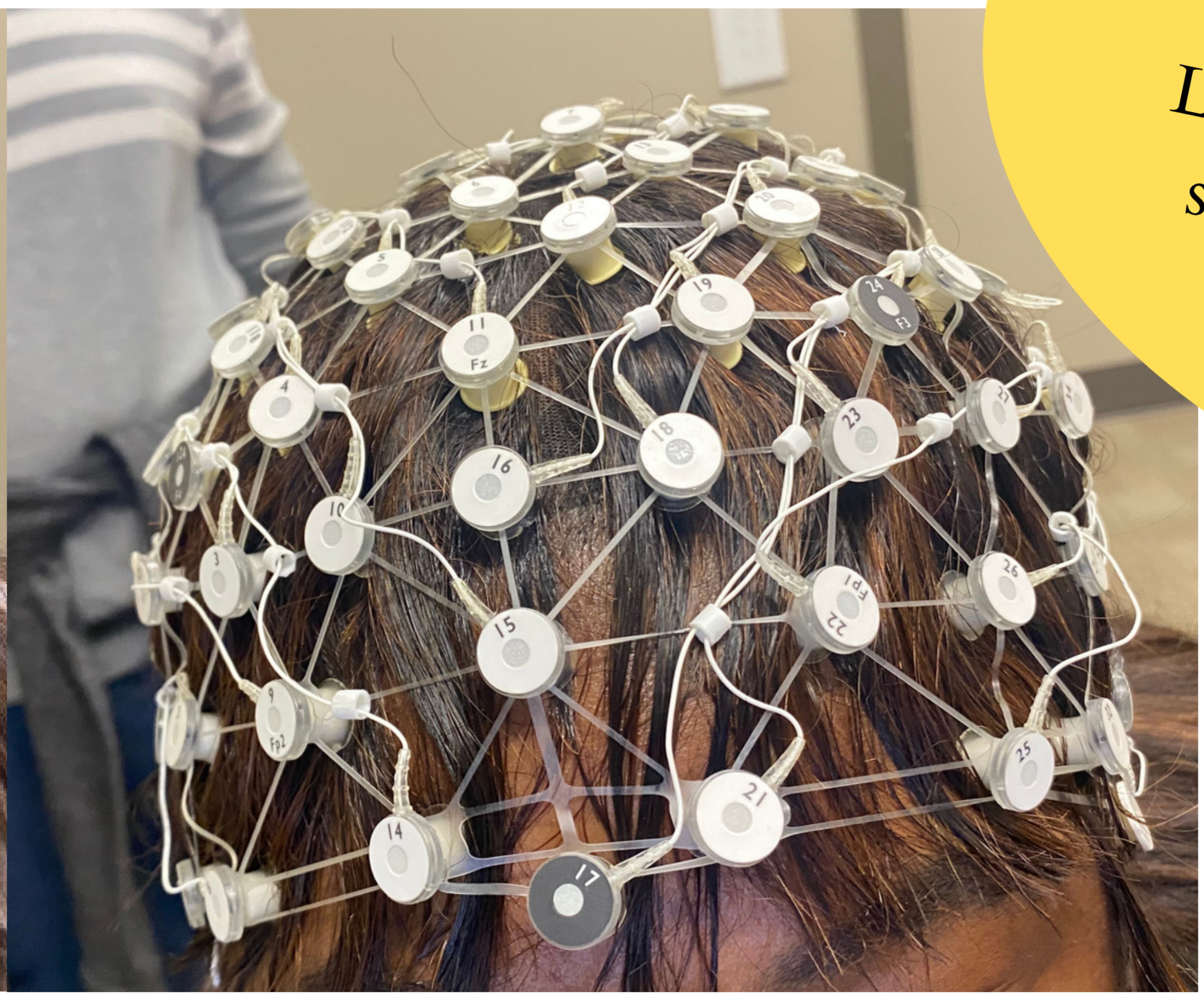

Do you have processed, freshly styled hair, or a wig that cannot get wet?

Let's chat about options! We can schedule before your next hair styling.

Long CURLS

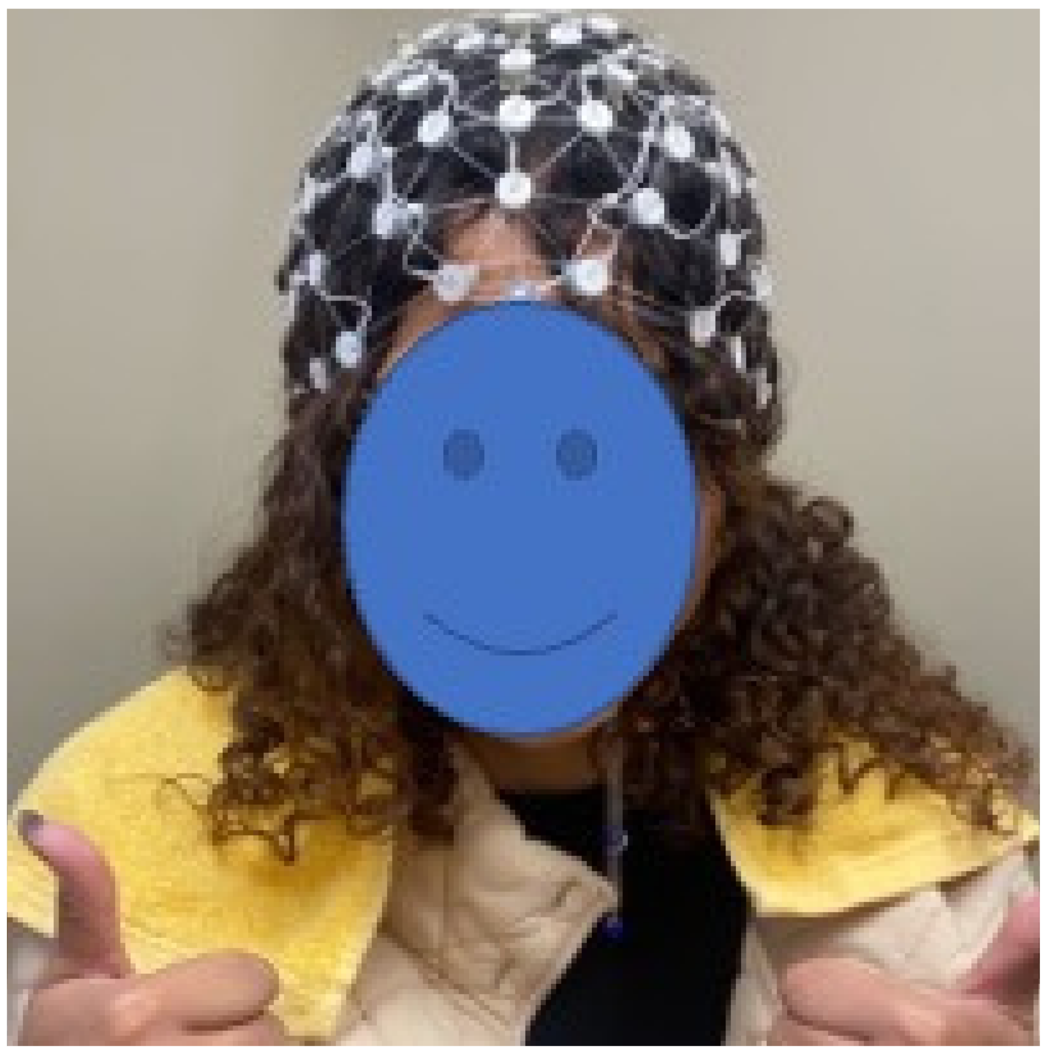

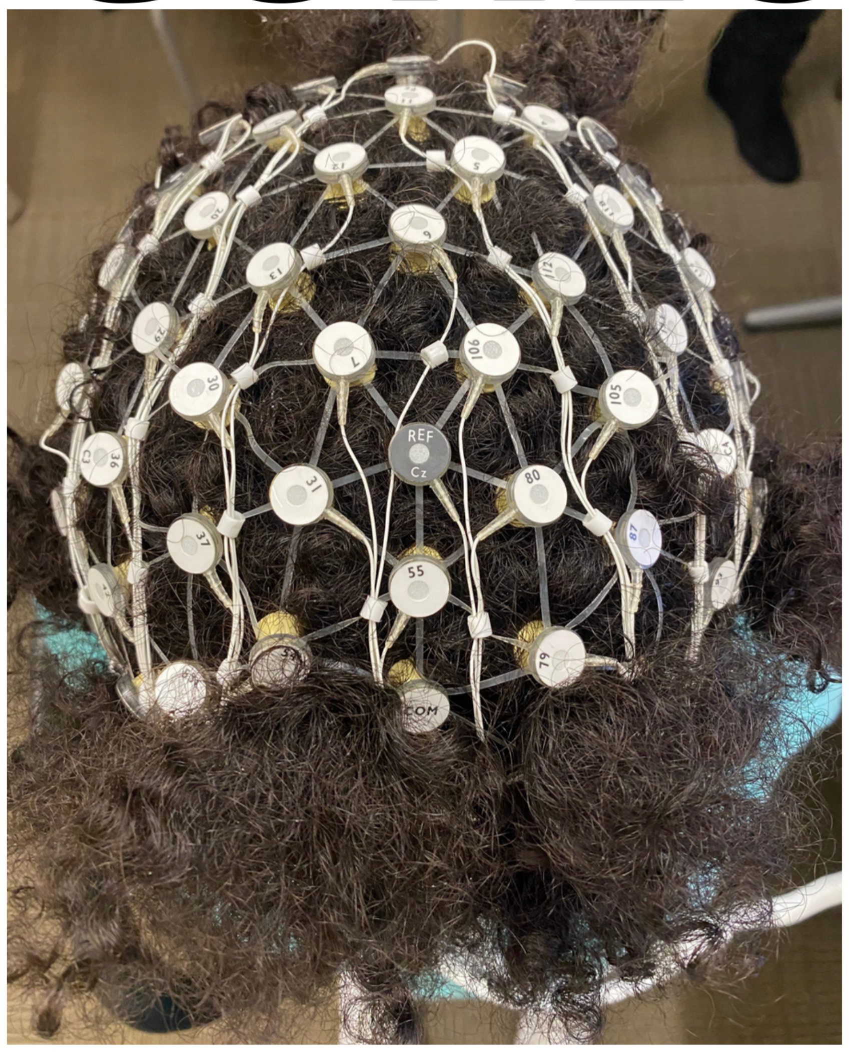

Developed by
Brain Research Across Development (B-RAD) Lab
& Dr. Caitlin Hudac

Contact us if you have questions!
b.rad.laboratory@gmail.com
