# Supplementary material for: Dynamic cognitive inhibition in the context of frustration: Increasing racial representation of adolescent athletes using mobile community-engaged EEG methods
Source: Front Neurol. 2022 Dec 21;13:918075. doi: 10.3389/fneur.2022.918075 (PMC9812645; doi:10.3389/fneur.2022.918075)
Supplement: Supplementary file 2 [file Presentation_2.pdf]

Strategies, procedures, and suggestions for applying Magstim-EGI Hydrocel geodesic sensor nets

Intended for researchers

Here, we describe methods adopted by our research team when using the Magstim-EGI Hydrocel geodesic sensor nets. We encourage users of other systems to prepare and share their methods for other systems/technologies.

We emphasize that our objective is to be inclusive to participants, above and beyond the strategies described here. These strategies are not meant to be exhaustive, but rather a foundation for individuals using this style of net with participants with coarse and curly hair. We encourage researchers to start conversations with each participant to determine their unique needs and answer questions.

Description of the Hydrocel nets. These nets are used within a high-impedance system. In the current version of the nets, the electrode is at the top of a 1 cm pedestal containing a sponge that is soaked in an electrolyte (potassium chloride) solution. The goal during net application is to ensure that the base of the pedestal is seated against the skin. Sensor wires are bundled and connected to an adaptor that connects to the amplifier. In order to place sensors at similar locations on the scalp (i.e. across sizes, sensors are connected to each other via an elastomer weaving. This is important, as the elastomer can stretch and break (see section below about sizing). It is important to maintain information about net "health" – we recommend monthly tests to inspect any elastomer stretching, electrodes that may have popped out of the netting, and an impedance check in water to determine if any electrodes will need to be replaced. We often replace or refurbish nets after ~100 uses and have found this rate is *not* different when accounting for participants with coarse, curly, and/or Afro-textured hair.

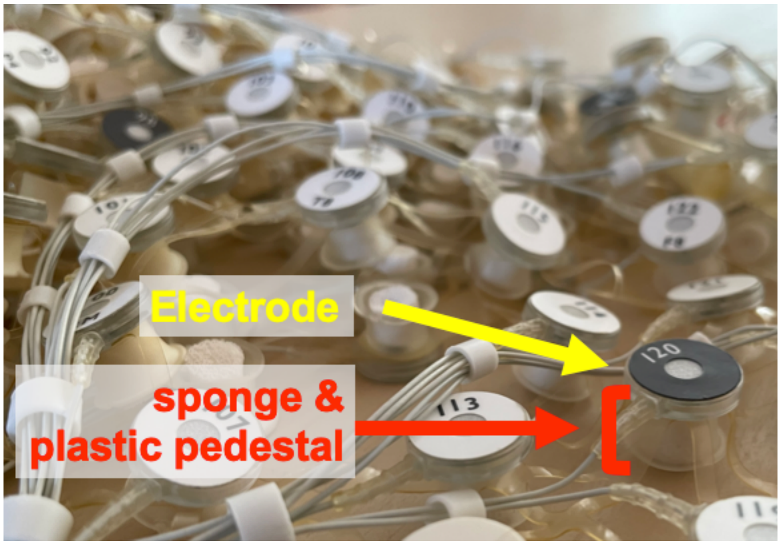

Electrolyte solution. We use the recommended amount of potassium chloride in our electrolyte solution. The manufacturers also recommend using baby shampoo to eliminate oils from the skin and hair. Depending on the hair texture, we may opt to use more baby shampoo. The goal is to soak the net for at least 10 minutes in the electrolyte solution. For Afro-textured hair, we recommend soaking for an additional 5-10 minutes if possible, ensure to keep a lid on the bucket to keep the solution warm for the participant's comfort.

Net sizing: Measure the head circumference as usual (nasion-inion). Be prepared to make net-size decisions (i.e., sizing up or down) dependent on your participant's hairstyle. For example, if your participant has thick locks or long Afro-textured hair, consider using a net one size bigger than the measured circumference to ensure a comfortable fit. Depending on the thickness of each braid, it may not be necessary to size up because we encourage researchers to pull individual braids through the netting. However, the initial net application should sit fairly comfortably on the participant's head.

Personnel and net application: It is recommended that two researchers apply the net. It is helpful that one researcher focuses on primary net placement from the front of the head ("front primary") while another researcher assists in successfully covering the back of the head with the net ("back secondary"). We use a technique where the front primary researcher reaches over the head so that the back secondary researcher can loop their fingers under the bottom row of the net against the back of the neck. Then, the back secondary researcher holds the net as a point of tension, so that the front primary researcher can place the net from the back to the front, making sure that it is correctly aligned (i.e., left to right) before securing the chin strap.

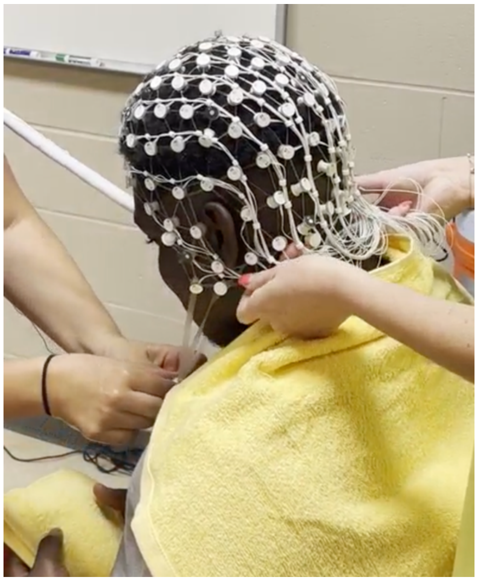

Figure 1: Front primary researcher tightening the chinstraps of the net while back secondary researcher pulls the back of the net down to ensure the net covers the entirety of the head

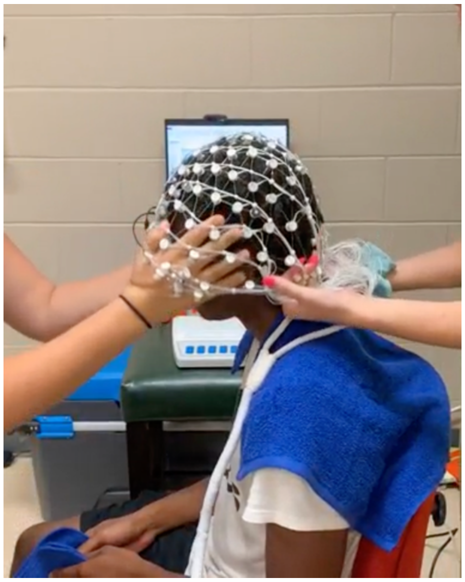

Figure 2: Front primary researcher pulling the net over the front of the head while back secondary researcher pulls the back of the net down to ensure full-net coverage

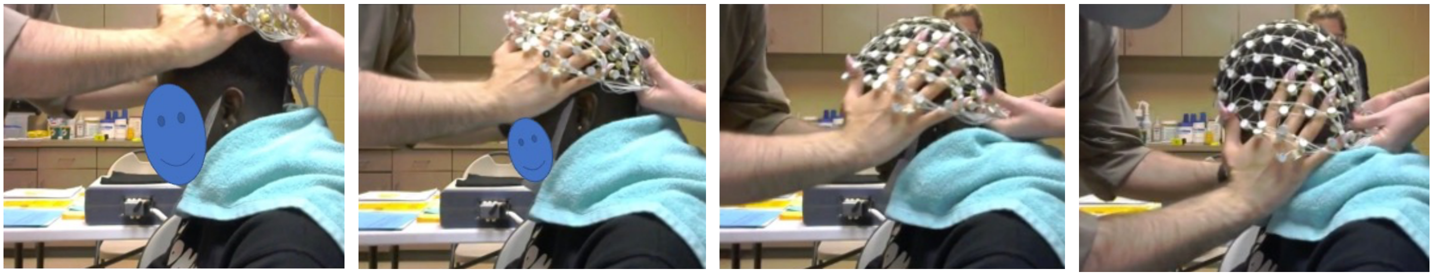

Figure 3: Video still frames of a net application (left-to-right) with two researchers.

Adjusting impedances: If impedances are high after net application, it can be important to verify why certain impedances are high. Are there electrode seating issues (i.e., thick hair)? Do the sponges need additional water? In terms of appropriately seating the electrodes, we have found strategies that work well: Try wetting the electrodes with a pipette and 'twisting' the electrodes in a back-and-forth circular motion to shift them underneath the hair and closer to the scalp. Do your best to divide roles based upon the skill points of your researchers (e.g., seating the electrodes, applying additional solution, "big picture" commands from someone watching the impedance window on the computer).

Hair Style: Keep hairstyle in mind when adjusting the net. Note that for hair styles such as twists, locks, and braids, if the hair is 'grown-out,' strands closer to the scalp may be more Afro-textured. In these cases, consider combining the above recommendations for the most-suitable net-application.

For participants with **longer locks or twists**, consider pulling the hair-strands through the elastomer weaving to bring the electrodes closer to the scalp. If too many strands of hair are pulled through the elastomer weaving on the sides, the center of the net may become loose. We recommend to first pull hair through at the vertex of the scalp before additional sections on the side. It can also be strategic for generating appropriate weight to keep electrodes seated on the scalp. You may consider prioritizing electrode-scalp contact for the electrodes in regions most crucial for your data collection.

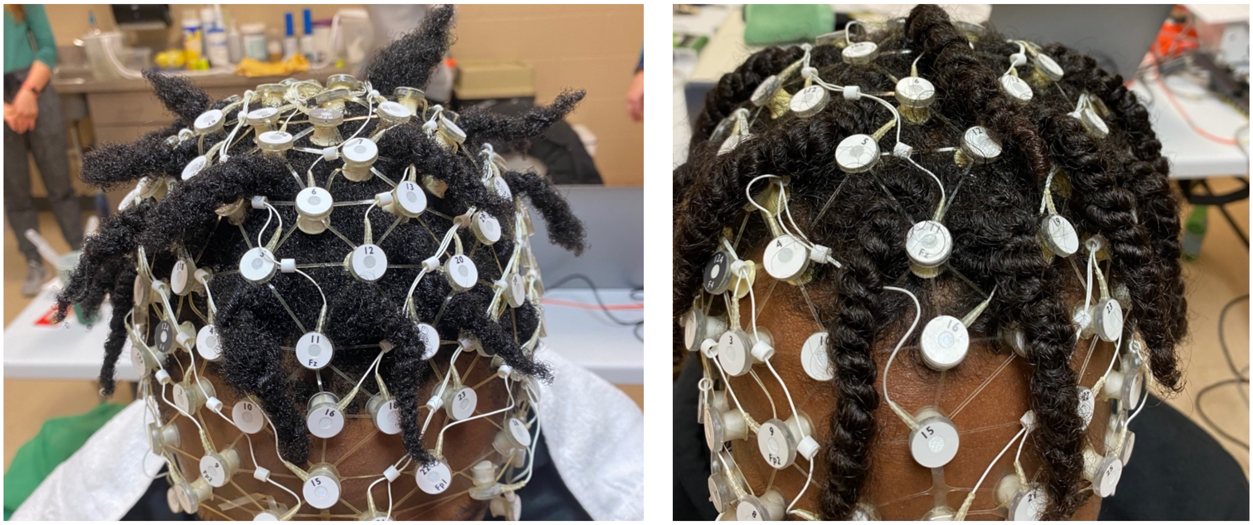

Figure 4: Examples of twists pulled through elastomer netting.

For participants with **Afro-textured hair**: start by 'wiggling' the EEG net 'down' into place, and if extra support is needed, consider applying a skull cap or durag overtop of the EEG net to maintain appropriate tension to aid in keeping the electrodes closer to the scalp:

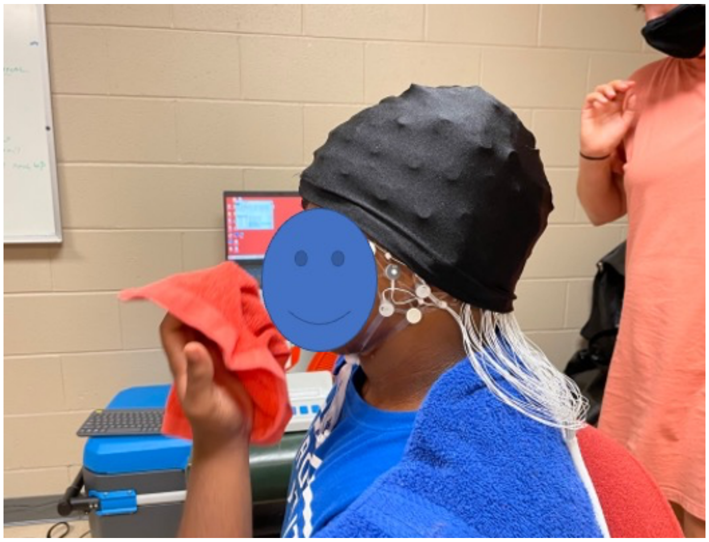

Figure 5: Examples of skull cap on top of the EEG net.

For participants with braids closer to the scalp (i.e., cornrows), adjust the net (front-back or left-right) to place the electrodes in-between the rows of braids rather than on top of the braids and consider using a gentle tape (i.e., medical tape) to hold the electrodes in place:

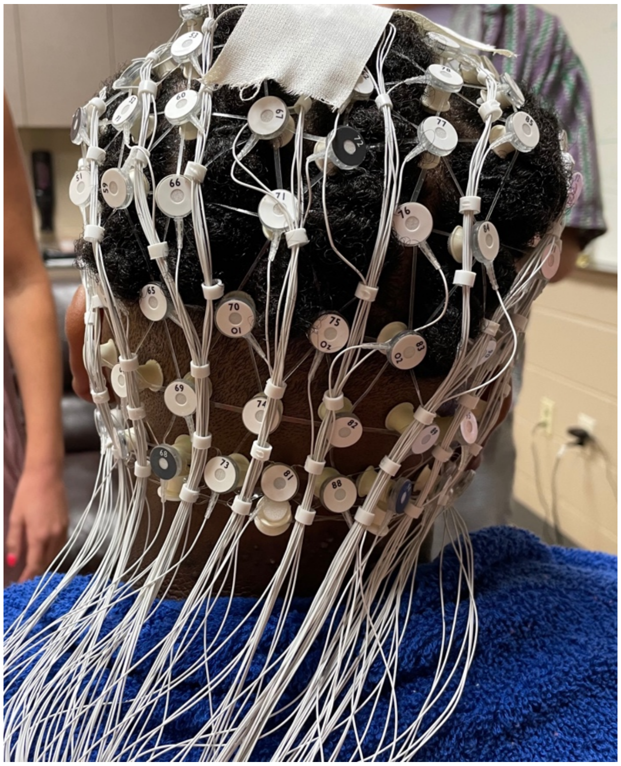

Figure 6: Examples of using medical tape to secure electrode placement.

Prioritized regions: Many times, researchers will have *a priori* regions of interest for their experimental tasks; thus, we encourage researchers to generate a plan for what to do if primary regions are covered by thick hair. It is possible to prioritize these regions, but this should be done in a systematic way and documented during acquisition. When using high-density systems, we also suggest generating a post-processing guide for how researchers will make decisions about placement deviations or other electrode inclusions (e.g., will you exclude rim electrodes, as is common in infant research?).

Flexibility and preparation: Try new things as needed! Be comfortable talking to participants about what they are comfortable with. If possible, communicate with your participant prior to their research session. It is helpful for participants to know whether the cap will be wet (i.e., in the case of processed hair) and if participation poses any risks to their current hair style. It also may be helpful to have a guide on hand to share with potential participants to ensure they are aware of what their participation may entail.

Additional frequently asked questions :

Can individuals with wigs participate in EEG research?
Yes! If your participant wears a wig, be sure to inquire about the wig adhesive. Some wig adhesives dissolve in water, so be sure that your participant knows their hair will be wet and is okay with it.

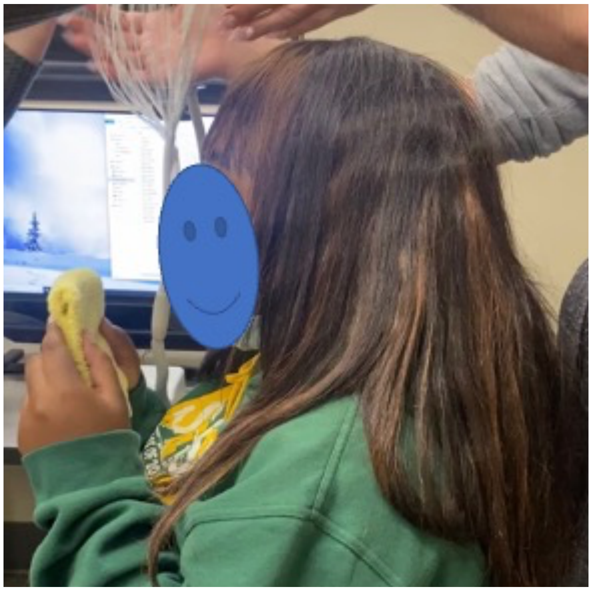

Figure 7: Participant with a lace glued wig before net application

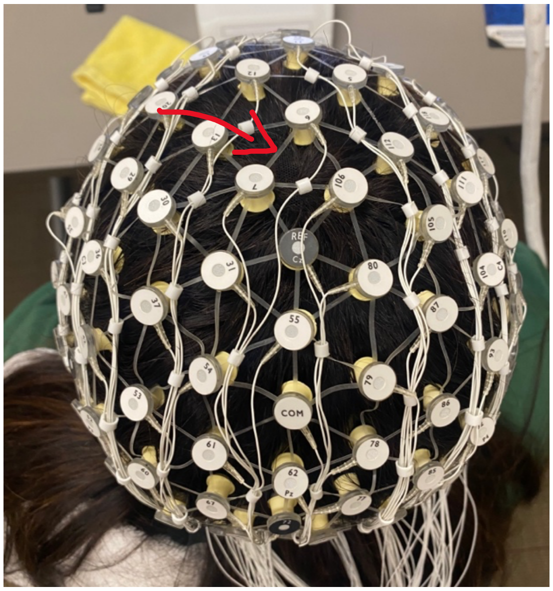

Figure 8: Participant with lace glued wig after net application. Notice the netting of the wig in-between the elastomers -- this did not affect our signal during data collection

What should you do if the reference electrode (e.g., vertex) is on a thick section of hair (e.g., braid or lock)?

Within our EEG acquisition, we record from the vertex electrode (C_z), which is occasionally an area that is covered by braids or locks. In our experience, it is better to adjust the net placement to ensure that the reference electrode is more likely to be well seated on top of the skin. We indicate deviations in net placement, take several photographs of the final net placement, and may make analytic decisions about how best to re-reference the electrical signal. Because we most often are utilizing an average reference during post-processing (i.e., transforming the data from a single reference point to an average of all electrodes), this has not negatively impacted the data quality. In extreme situations, it may be important to be more individually-selective about electrodes in the analysis. When possible, using a geo-positional system, as is custom in source estimation, to photograph and record net sensors can be helpful.

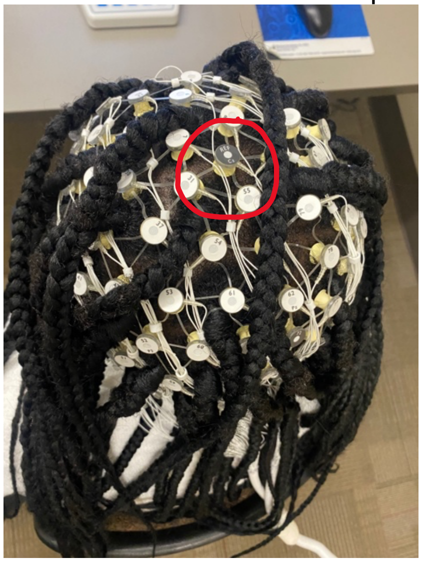

Figure 9: Slightly shift the electrode to the base of the braid or lock while still staying as close to the vertex (where the vertical and horizontal hemispheres cross) of the head as possible.

Can individuals with sew-in hair extensions participate in EEG research?
Yes! If your participant has sewn-in hair extensions, be mindful of the track (i.e., horizontal sewn area) and/or seam (i.e., bonded bundle) of the extensions. Locate where the extensions are beaded, braided, glued, or taped to the existing hair and strategically arrange the electrodes around this area to produce the best possible signal.

To find the seam or track of the extensions: with your participant's permission, place your hand on the back of their head until you feel a 'bulge' underneath their hair. The track or seam may be one straight line across the head, multiple straight lines across the head, a semi-circle-shaped line surrounding the center of the head, etc. After net application, place the electrodes on either side of the seam(s)/track(s). If extra support to hold the electrodes in place is needed, consider using a gentle tape (i.e., medical tape) to hold the electrodes in place.

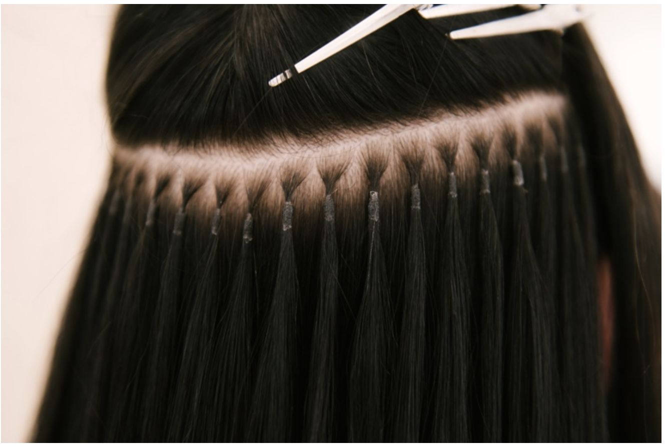
Figure 10: An example of hair-extension tracks.
